# Supplementary material for: Splice-Junction-Based Mapping of Alternative Isoforms in the Human Proteome
Source: Cell Rep. Author manuscript; Available in PMC 2020 Jan 15. (PMC6961840; doi:10.1016/j.celrep.2019.11.026)
Supplement: 2 [file NIHMS1546469-supplement-2.zip › DF1/PXD010154/Liver/Liver_61_LRRC28_KPCSEASKPCGTVELELR.pdf]

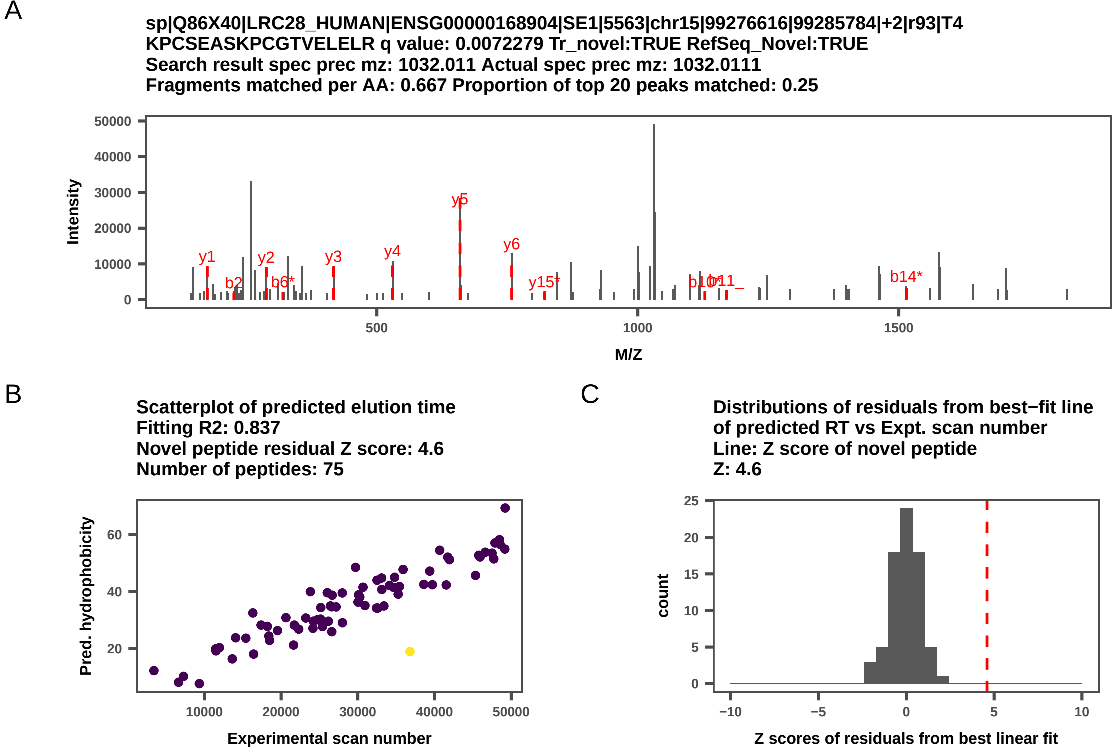
A
sp|Q86X40|LRC28_HUMAN|ENSG00000168904|SE1|5563|chr15|99276616|99285784|+2|r93|T4
KPCSEASKPCGTVELELR q value: 0.0072279 Tr_novel:TRUE RefSeq_Novel:TRUE
Search result spec prec mz: 1032.011 Actual spec prec mz: 1032.0111
Fragments matched per AA: 0.667 Proportion of top 20 peaks matched: 0.25
Intensity
50000
40000
30000
20000
10000
0
y1
b2
y2
b6*
y3
y4
y5
y6
y15*
b10*
b11_
b14*
500
1000
1500
M/Z
B
Scatterplot of predicted elution time
Fitting R2: 0.837
Novel peptide residual Z score: 4.6
Number of peptides: 75
Pred. hydrophobicity
60
40
20
10000
20000
30000
40000
50000
Experimental scan number
C
Distributions of residuals from best−fit line
of predicted RT vs Expt. scan number
Line: Z score of novel peptide
Z: 4.6
count
25
20
15
10
5
0
−10
−5
0
5
10
Z scores of residuals from best linear fit
